# Likelihood representation in the owl's sound localization system

**DOI:** 10.1186/1471-2202-14-S1-P128

**Published:** 2013-07-08

**Authors:** Fanny Cazettes, Brian J Fischer, José L Peña

**Affiliations:** 1 Department of Neuroscience, Albert Einstein College of Medicine, Bronx, New York, 10461, USA; 2 Department of Mathematics, Seattle University, Seattle, Washington, 98122, USA

## 

The tug of war between expectation and evidence is an intricate problem in perceptual judgments. The Bayesian framework provides a probabilistic solution to this problem. It describes how sensory information, represented by a likelihood function, is combined with previous knowledge of the world, known as the prior distribution, to generate optimal behavioral outputs. Thus, the shape of both the likelihood and the prior determine whether neural representations obey optimal coding. Yet, how the brain represents likelihood functions is unknown. Here, we address this question in the auditory system of barn owls, which displays a map of space in the external nucleus of the inferior colliculus (ICx) [1,2]. Due to their non-ambiguous tuning to interaural time differences (ITD), a critical spatial auditory cue, ICx neurons are selective to horizontal direction [3,4]. This selectivity arises from the convergence of frequency channels that originate in lower-brainstem ITD-detector cells narrowly tuned to frequency [5-7]. If ICx neurons implement Bayesian inference, spatial tuning in ICx should conform to the relationship between sound direction and ITD specified by the likelihood. The proposed likelihood model predicts that the widths of the tuning curves should increase with eccentricity [8]. Because the width of ITD tuning curves is determined by the preferred frequency of the cells [9], we hypothesize that non-uniform frequency convergence is involved in non-uniform spatial tuning. Yet, the pattern of frequency convergence across space has never been linked to the spatial tuning widths. Interestingly, theory [8] predicts that ITD tuning across frequency should also be determined by the filtering properties of the head.

We investigate how frequency convergence governs ITD integration across frequency to understand the physiological representation of likelihood. We examine the tuning properties of midbrain neurons using *in vivo *extracellular recordings. The preferred ITDs and ITD tuning curve widths were measured at fine resolution in ICx. Consistent with our hypothesis, we found that the non-uniform ITD tuning predicted by the likelihood model is not present at stages in the ITD pathway where neurons do not integrate across frequency. We have also examined the relationship between frequency tuning and ITD tuning. Importantly, we found that the distribution of preferred frequency in ICx also depends on ITD tuning. We are currently testing if the representation of likelihood relies on the filtering properties of the head by assessing whether ITD tuning varies across frequency and space.

The experimental results are combined to model how the network represents the statistical dependence of ITD on sound direction. The input signals to the ears are first filtered with a gammatone filter. ITD curves are then extracted from the output of the filterbank using a cross-correlation operation [7]. ITD-curves are merged with different weights estimated from the data and a linear-nonlinear Poisson model is used to describe the responses in ICx. With our model we can parse out the importance of each mechanism in the representation of likelihood.
